# Incidence and factors on anaemia during pregnancy in China: a multicentre prospective cohort study

**DOI:** 10.7189/jogh.16.04058

**Published:** 2026-02-13

**Authors:** Xiaosong Zhang, Xueyin Wang, Juan Juan, Di Gao, Huixia Yang, Meihua Zhang, Xu Chen, Xietong Wang, Yuyan Ma, Yue Teng, Guohua Zhang, Yaqin Wang, Haixia Meng, Xiaoqing Wang, Qiuhong Yang, Lin Xu, Shufan Shan

**Affiliations:** 1 Obstetrics and Gynaecology Department of Peking University First Hospital, Beijing, China; 2 Obstetrics Department of Taiyuan Maternity and Child Care Hospital, Taiyuan, China; 3 Obstetrics Department of Tianjin Central Hospital of Gynaecology Obstetrics, Tianjin, China; 4 Obstetrics and Gynaecology Department of Shandong Provincial Maternal and Child Health Care Hospital Affiliated with Qingdao University, Jinan, China; 5 Obstetrics and Gynaecology Department of Qilu Hospital of Shandong University, Jinan, China; 6 Nutrition Department of Haidian District Maternal and Child Health Care Hospital, Beijing, China; 7 Obstetrics Department of Shijiazhuang Obstetrics and Gynaecology Hospital, Shijiazhuang, China; 8 Obstetrics and Gynaecology Department of Shaanxi Provincial People’s Hospital, Xi’an, China; 9 Obstetrics Department of The Affiliated Hospital of Inner Mongolia Medical University, Hohhot, China; 10 Obstetrics Department of the Third People’s Hospital of Datong, Datong, China; 11 Obstetrics Department of Jinan Maternity and Child Care Hospital Affiliated with Shandong First Medical University, Jinan, China; 12 Obstetrics Department of The Affiliated Hospital of Qingdao University, Qingdao, China; 13 Obstetrics Department of the Affiliated Hospital of Chifeng University, China, China

## Abstract

**Background:**

As anaemia during pregnancy is common in China, we sought to investigate the incidence and influencing factors on maternal anemia in the northern part of China.

**Methods:**

We conducted a prospective cohort study from October 2020 to June 2024 at 13 hospitals in China, enrolling 18 416 pregnant women aged ≥18 years who had regular prenatal examinations, Their data (location, history of anaemia, and haemoglobin (Hb) test results) were collected *via* an app data collection platform. They were enrolled after providing informed consent in the first trimester at 11–13 weeks, and followed up at 24–27 gestational weeks, 32–35 gestational weeks, and ≥36 weeks. They provided information independently and uploaded their laboratory test results in the form of photographs at each follow up. Univariate and multivariate logistic regression models were used to analysis.

**Results:**

After excluding 7431 participants with incomplete Hb data, we were left with 10 985 pregnatn women who had taken four Hb tests in each interview at 11 hospitals in five provinces. The overall prevalence of maternal anaemia was 23.2% (95% CI = 22.5–24.0%). Its overall incidence in the second or third trimester was 20.5% (95% CI = 19.8–21.3%) and was highest at 24–27 weeks (13.8%, 95% CI = 13.1–14.4%). Factors influencing incident maternal anaemia were parity ≥2 times (OR = 2.1; 95% CI = 1.51–2.92), a history of anaemia before pregnancy (OR = 1.77, 95% CI = 1.41–2.23), and a history of anaemia during a previous pregnancy (OR = 1.71, 95% CI = 1.37–2.13). We observed a positive association between pre-pregnancy BMI categorised as overweight (BMI = 24–27.9: OR = 0.68; 95% CI = 0.59–0.77) or obesity (BMI ≥28: OR = 0.79; 95% CI = 0.67–0.94) with maternal anaemia.

**Conclusions:**

Both of prevalence and incidence of anaemia in pregnancy were higher in the second trimester. Targeted action is needed for women with a history of anaemia before or during a previous pregnancy and women who had multiple deliveries. Such efforts could include strengthened screening programs or better health education on prenatal nutrition during pregnancy and pre-pregnancy.

**Registration:**

ClinicalTrials.gov: NCT04486456.

Anaemia during pregnancy is a common maternal problem; in 2019, its prevalence in women aged 15–49 in 2019 was estimated to be 36%, with drastically higher rates in high-income (15%) compared to lower-income countries in western and central Africa (52%) [1,2]. Anaemia during pregnancy is associated with an increased risk of adverse birth and health outcomes, postpartum haemorrhage, placental abruption, maternal shock, and consequent intensive care unit admission. Moreover, it increases the risks of low birth weight, preterm birth, perinatal mortality, neonatal mortality and neurological damage to the child [3–6].

Improvements in China’s socioeconomic status and healthcare system in the past 70 years have also led to better health for children and mothers alike [7]. However, anaemia during pregnancy remains one of the main pregnancy-related complications in China, with a recent meta-analysis of 36 studies showing its pooled prevalence to be 30.7% (95% CI = 26.6–34.7%), albeit with sizable regional variation and a lower prevalence in eastern and urban areas, possibly due to differences in economic status and populations’ dietary behaviours [8]. Other studies have shown socioeconomic factors, maternal age, and high parity to be some of the factors affecting the development of anaemia during pregnancy [8–11]. Therefore, to achieve the goal of lowering the prevalence of maternal anaemia below 10% by 2030 [12], China must implement screening programmes to identify high-risk women on time, before the occurrence of anaemia. However, most studies on maternal anaemia in the country were cross-sectional in design, with data on the incidence in each of the trimesters being especially lacking. Therefore, in this multicentre prospective cohort study, we sought to determine the incidence of anaemia during pregnancy at different gestational time points and the associated risk factors.

## METHODS

### Time and sites

We conducted a prospective cohort study from October 2020 to June 2024 in 13 hospitals in seven provinces in North China: Beijing, Tianjin, Shannxi Province, Shanxi province, Shandong Province, Inner Mongolia, and Hebei Province.

### Participants

We included pregnant women who had regular prenatal examinations and planned to be hospitalised for delivery, provided they were aged ≥18 years and consented to participant in the survey by signing an informed consent form. We excluded women with a pre-pregnancy history of severe chronic diseases or pregnant women with severe mental illness. The relative prevalence of maternal anaemia in Chinese hospitals was found to be 19.8% [13]. In PASS 15 (NCSS, LLC., Kaysville, Utah, USA), assuming an α of 0.05 and a power of ≥90%, we calculated the required sample size to be 9661 participants.

### Data collection process

We enrolled all eligible pregnant women in the first trimester at 11–13 weeks and followed them up three times in the second (24–27 gestational weeks (GWs)) and third (32–35 GWs) trimester, and once at ≥36 weeks. We collected their geographical location, history of anaemia, and Hb test results using the ‘91trial’ application, an electronic data collection platform we developed specifically for this project. In the first interview at 11–13 GWs, professional staff assisted the pregnant women download the ‘91trial’ application and share their data, as well as upload photographs of their laboratory test results. They also provide the following sociodemographic data at this point: age, nationality (Han or minority), employment status (‘yes’ or ‘no’), educational level (junior high school or below, senior high school, and university or above), family income/month (RMB<3000, 3000–5000, 5000–10 000, and >10 000). The clinical characteristics included pre-pregnancy body mass index (BMI), calculated as kg/m^2^ and categorised as underweight (<18.5), normal (18.5–23.9), overweight (24–27.9), obesity (≥28), according to the Chinese standard [14]; gravidity ; parity (0, 1 and ≥2); history of anaemia (‘yes’ or ‘no’); and history of anaemia in previous pregnancies (‘yes’ or ‘no’). Other information included smoking before pregnancy (‘yes’ or ‘no’), exposure to second-hand smoke before pregnancy (‘yes’ or ‘no’), alcohol consumption before pregnancy (‘yes’ or ‘no’), the frequency of coffee consumption (‘everyday’, ‘1–2 days/week’, or ‘never’), the frequency of tea consumption (‘every day’, ‘1–2 days/week’, or ‘never’), and use of supplemental iron (‘yes’ or ‘no’).

The participants then provided their haemoglobin (Hb) values on their own at the three remaining time points, with reminders being sent to them to complete the survey. As all the study hospitals were tertiary hospitals with laboratory department overseen regularly in terms of quality control by the National centre for Clinical Laboratories, all laboratory tests were likewise conducted according to same standards and criteria.

### Criteria for the definitions of anaemia and outcome variables

**Figure 1:**
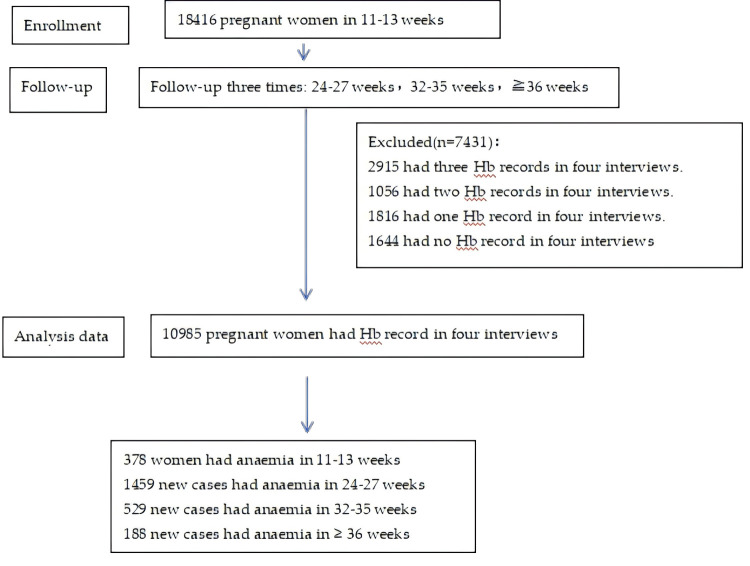
Flowchart showing the data of the pregnant women enrolled in the study.

We defined anaemia as a Hb values of <110 g/L and further categorised it as follows: mild anaemia (100–109 g/L), moderate anaemia (70–99 g/L) and severe anaemia (Hb <70 g/L) [15]. We calculated the overall incidence of anaemia, as follows: the numerator were the new cases of participants with anaemia at 24–27, 32–35, or ≥36 weeks, while the denominator was the number of women without anaemia at 11–13 weeks (**Figure 1**).

### Statistics

We presented categorical variables as numbers and percentages. Using the Kolmogorov-Smirnov test and Q-Q plots, we checked the normality of distribution for continuous variables; if it was normal, we presented them as means and standard deviations (SDs). We constructed univariate logistic regression models to assess the associations between the incidence of anaemia during the second or third trimester and anaemia. We also used multivariate logistic regression models to analyse the associations between the incidence of anaemia during pregnancy and other variables, with the models either unadjusted or adjusted for variables that were statistically significant in the univariate logistic regression models (age, education, BMI before pregnancy, history of anaemia, history of anaemia in previous pregnancies, history of gestation, and parity), and presented their outputs as odds ratios (ORs) and 95% confidence intervals (CIs). We performed multiple imputation for participants with missing maternal age (n = 869). We performed all analyses in SPSS, version 26 (IBM, Armonk, New York, USA). All *P*-values are two-sided, with values <0.05 indicating statistical significance. 

### Ethics

The Biomedical Research Ethics Committee of Peking University First Hospital (2018 (267)) approved this study. All women provided informed consent before enrolment. The full study protocol is on ClinicalTrials.gov (NCT04486456).

## RESULTS

In total, 18 416 pregnant women attended the first interview at 13 hospitals, with 10 895 uploading all four Hb tests at 11 hospitals in five provinces: Beijing, Tianjin, Shanxi Province, Shandong Province, and Inner Mongolia (**Figure 1**). The average age at pregnancy was 31.4 (standard deviation = 4.1, range = 18–51) years, with just below three fourths of women being 26–34 year old (**Table 1**).

**Table 1:** Sociodemographic and clinical characteristics of the participants, n (%)

	Participants (n = 10 985)
**Age in years**	
≤25	694 (6.3)
26–34	7905 (72)
≥35	2386 (21.7)
**Nationality**	
Han	10 584 (96.3)
Minority	401 (3.7)
**Employment**	
Yes	9665 (88)
No	1320 (12)
**Educational level**	
Junior high school or below	438 (4)
Senior high school	818 (7.4)
University or above	9729 (88.6)
**Family income/month in RMB**	
<3000	351 (3.2)
3000–5000	1056 (9.6)
5000–10 000	3603 (32.8)
>10 000	5975 (54.4)
**History of anaemia**	
Yes	473 (4.3)
No	10 512 (95.7)
**Gravity**	
0	6479 (59)
1	2560 (23.3)
≥2	1946 (17.7)
**Parity**	
0	8216 (74.8)
1	2521 (22.9)
≥2	248 (7.3)
**Pre-pregnancy BMI**	
<18.5	957 (8.7)
18.5–23.9	6775 (61.7)
24–27.9	2251 (20.5)
≥28	1002 (9.1)
**History of anaemia in previous pregnancies**	
Yes	535 (4.9)
No	10 450 (95.1)
**Smoking before pregnancy**	
Yes	156 (1.4)
No	10 829 (98.6)
**Second-hand smoking before pregnancy**	
Yes	741 (6.7)
No	10 244 (93.3)
**Alcohol consumption**	
Yes	724 (6.6)
No	10 261 (93.4)
**Coffee consumption**	
Everyday	496 (4.5)
1–2 days/week	1250 (11.4)
No	9239 (84.1)
**Tea consumption**	
Everyday	888 (8.1)
1–2 days/week	1767 (16.1)
No	8330 (75.8)
**Iron supplementation**	
Yes	3630 (33)
No	7355 (67)

### Prevalence of anaemia according to the GW of pregnancy

**Figure 2:**
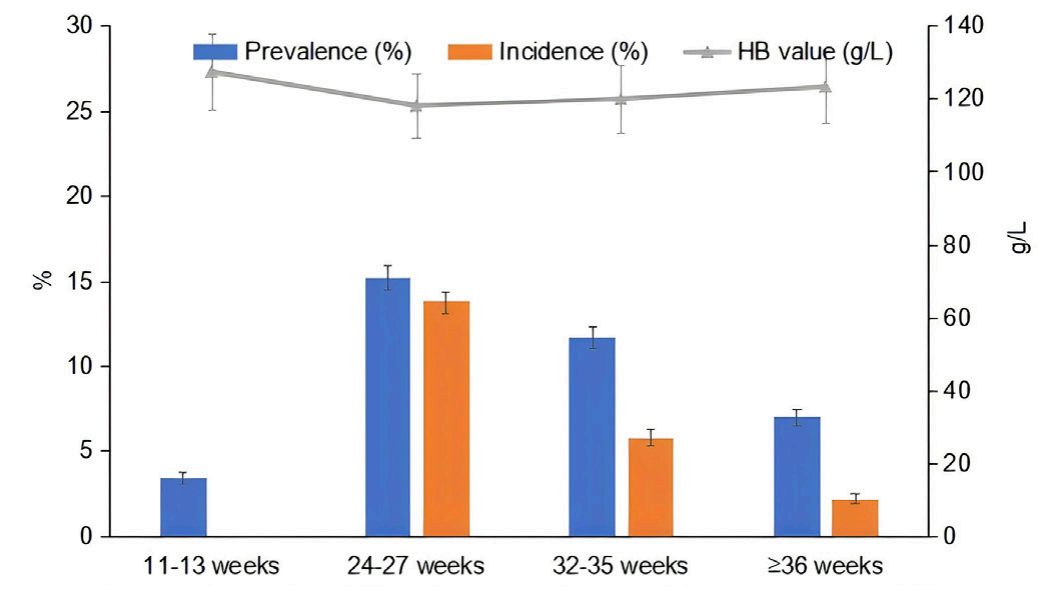
Prevalence and incidence rate of anaemia and Hb values at different GWs.

The mean Hb values were 127.35 g/L (SD = 10.34, range = 62–167) at 11–13 GWs, 118.02 g/L (SD = 8.86, range = 76–165) at 24–27 GWs, 119.87 g/L (SD = 9.26, range = 68–160) at 32–35 GWs, and 123.20 g/L (SD = 9.94, range = 69–167) at ≥36 GWs (**Figure 2**). In total, 378 pregnant women had anaemia at the first antenatal care interview, amounting to a prevalence of 3.4% (95% CI = 3.1–3.8%). The prevalence was the highest at 24–27 GWs (15.2%, 95% CI = 14.5–15.9%), followed by 32–35 GWs (11.7%; 95% CI = 11.1–12.3%) and ≥36 GWs (7.0%; 95% CI = 6.5–7.5%), respectively (**Figure 2**). The overall prevalence of anaemia during pregnancy was 23.2% (95% CI = 22.5–24.0%), and most anaemia cases were mild (**Table 2**).

**Table 2:** Anaemia categories according to GWs, n (%)

Anaemia category	11–13 weeks	24–27 weeks	32–35 weeks	≥36 weeks
Normal	10 607 (96.6)	9315 (84.8)	9697 (88.3)	10 214 (92.9)
Mild	286 (2.6)	1443 (13.1)	1120 (10.2)	665 (6.1)
Moderate	90 (0.8)	227 (2.1)	167 (1.5)	105 (1.0)
Severe	2 (0.0)	0 (0.0)	1 (0.0)	1 (0.0)
Total	10 985 (100)	10 985 (100)	10 985 (100)	10 985 (100)

### Incidence of anaemia according to the GW

In the first antenatal interview at 11–13 GWs, 10 607 pregnant women did not have anaemia. The incidence at 24–27 GWs (n/N = 1459/10 607), 32–35 GWs (n/N = 529/9148), and beyond 36 GWs (n/N = 188/8619) was 13.8% (95% CI = 13.1–14.4%), 5.8% (95% CI = 5.3–6.3%), and 2.2% (95% CI = 1.9–2.5%), respectively. The incidence in the second or third trimester (n/N = 2176/10 607) was 20.5% (95% CI = 19.8–21.3%) (**Figure 1**, **Figure 2**).

### Factors associated with the incidence of anaemia during the second or third trimester

In the multivariable logistic regression analysis (**Table 3**), the factors influencing incident anaemia during pregnancy were parity ≥2 times (OR = 2.1; 95% CI = 1.51–2.92), a history of anaemia before pregnancy (OR = 1.77; 95% CI = 1.41–2.23), and a history of anaemia during a previous pregnancy (OR = 1.71, 95% CI = 1.37–2.13). We also found positive associations of pre-pregnancy BMI categorised as overweight (OR = 0.68; 95% CI = 0.59–0.77) or obesity (OR = 0.79; 95% CI = 0.67–0.94) with anaemia during pregnancy.

**Table 3:** Associations between risk factors and the incidence of anaemia in the second or third trimester

	Model 1*		Model 2†	
	**OR (95% CI)**	***P*-value**	**OR (95% CI)**	***P*-value**
**Age in years**				
26–34	ref		ref	
≤25	1.09 (0.90–1.33)	0.369	1.12 (0.92–1.37)	0.260
≥35	1.17 (1.04–1.30)	0.008	1.09 (0.97–1.23)	0.164
**Ethnicity**				
Han	ref		ref	
Minority	1.06 (0.83–1.35)	0.648	1.04 (0.81–1.34)	0.745
**Employment**				
Yes	ref		ref	
No	1.03 (0.89–1.19)	0.686	0.98 (0.84–1.15)	0.822
**Educational level**				
Junior high school or below	ref		ref	
Senior high school	0.79 (0.59–1.05)	0.105	0.82 (0.61–1.09)	0.174
University or above	0.79 (0.62–0.99)	0.045	0.83 (0.65–1.04)	0.153
**Family income/month in RMB**				
<3000	ref		ref	
3000–5000	0.97 (0.72–1.34)	0.927	1.05 (0.77–1.44)	0.751
5000–10 000	0.84 (0.63–1.12)	0.239	0.90 (0.67–1.20)	0.460
>10 000	1.07 (0.81–1.41)	0.644	1.08 (0.81–1.44)	0.611
**BMI before pregnancy**				
Normal (18.5–23.9)	ref		ref	
Underweight (<18.5)	1.14 (0.97–1.34)	0.117	1.16 (0.98–1.36)	0.085
Overweight (24–27.9)	0.68 (0.60–0.78)	<0.001	0.68 (0.59–0.77)	<0.001
Obesity (≥28)	0.83 (0.70–0.98)	0.032	0.79 (0.67–0.94)	0.009
**Gravidity**				
0	ref		ref	
1	1.12 (0.99–1.25)	0.052	0.98 (0.84–1.13)	0.734
≥2	1.31 (1.16–1.49)	<0.001	0.98 (0.82–1.17)	0.800
**Parity**				
0	ref		ref	
1	1.28 (1.14–1.42)	<0.001	1.17 (0.99–1.38)	0.065
≥2	2.44 (1.85–3.20)	<0.001	2.10 (1.51–2.92)	<0.001
**History of anaemia before pregnancy**				
No	ref		ref	
Yes	2.15 (1.75–2.63)	<0.001	1.77 (1.41–2.23)	<0.001
**History of anaemia in previous pregnancies**				
No	ref		ref	
Yes	2.25 (1.85–2.73)	<0.001	1.71 (1.37–2.13)	<0.001
**Smoking**				
No	ref		ref	
Yes	1.07 (0.72–1.57)	0.745	1.03 (0.67–1.55)	0.885
**Second-hand smoking before pregnancy**				
No	ref		ref	
Yes	0.98 (0.81–1.19)	0.858	0.96 (0.79–1.16)	0.656
**Alcohol consumption**				
No	ref		ref	
Yes	1.02 (0.84–1.23)	0.861	0.98 (0.80–1.20)	0.876
**Coffee consumption**				
Never	ref		ref	
Daily	0.94 (0.81–1.09)	0.387	1.12 (0.89–1.42)	0.333
1–2 days/week	1.07 (0.83–1.38)	0.595	1.06 (0.90–1.42)	0.505
**Tea consumption**				
Never	ref		ref	
Daily	1.02 (0.86–1.12)	0.788	0.93 (0.77–1.12)	0.415
1–2 days/week	1.01 (0.89–1.5)	0.858	0.98 (0.84–1.12)	0.676
**Iron supplementation**				
No	ref		ref	
Yes	1.01 (0.92–1.12)	0.799	1.00 (0.91–1.11)	0.949

*Unadjusted.

†Adjusted for age, ethnicity, education, employment, family income/month, pre-pregnancy BMI, history of anaemia, history of anaemia in previous pregnancies, gravidity and parity, smoking, second-hand smoking, alcohol consumption, tea and coffee consumption, and iron supplements.

## DISCUSSION

We found the prevalence of prevalence of anaemia during pregnancy in our sample to be 3.4% at 11–13 GWs, 15.2% at 24–27 GWs, 11.7% at 32–35 GWS, and 7.0% at ≥36 GWs. The overall prevalence of anaemia during pregnancy was 23.2%; its overall incidence in the second or third trimester was 20.5%, and was highest at 24–27 GWs (13.8%). Parity ≥2 times, a history of pre-pregnancy anaemia, and a history of anaemia in previous pregnancies were influencing factors for anaemia in the second or third trimester, while overweight and obesity before pregnancy also had positive association with of anaemia.

The prevalence of maternal anaemia varies across countries and regions. One study found the prevalence of anaemia in pregnant women aged 15–49 years to be 36% at the global level, 15% in high-income countries, and 27% in East China and Southeast Asia [2]. Other studies found the prevalence of maternal anaemia to be 26.7% in the USA [16], 31.3% in South Africa [17], and 34.8% in northwest China [18]. A meta-analysis of 36 studies in China estimates the national prevalence of anaemia during pregnancy to be 30.7% [8]. The prevalence rates we identified here were similar to those reported in the studies from the USA [16], East and Southeast Asia [2], but lower compared to other studies in China [8,18]. This could reflect the fact that our research hospitals were located in urban areas with better economic conditions, which could explain our prevalence rate being closer to that of developed areas.

We also found the mean Hb values to be 127.35 g/L (SD = 10.34) at 11–13 GWs, 118.02 g/L (SD = 8.86) at 24–27 GWs, 119.87 g/L (SD = 9.26) at 32–35 GWs, and 123.20 g/L (SD = 9.94) at ≥36 GWs. This finding aligns with the results of an USA-based study, in which the average Hb values were 12.6 g/dl (SD = 1.0) in the first trimester, 11.6 g/dl (SD = 1.1) in the second trimester, and 11.8 g/dl (SD = 1.3) in the third trimester [16]. In our study, the incidence in the second or third trimester was 20.5% among pregnant women who did not have anaemia in the first trimester, with the incidence at 24–27 GWs being the highest. One of the main causes of anaemia during pregnancy is physiological haemodilution. A systematic review reported that the plasma volume grows by 50% during pregnancy; specifically, it begins increasing as early as in the first trimester of pregnancy, hikes steeply during the second trimester, and continues to rise until term [19,20]. Anaemia in pregnant individuals is mostly associated with iron deficiency, which results from increased iron demand during foetal growth, placental development, and maternal blood volume expansion. In total, approximately 1 g of additional iron is needed to sustain a healthy pregnancy over the course of gestation. The demand further increases as pregnancy progresses from approximately 1 mg/d in non-pregnant females to nearly 7 mg/d in the third trimester of pregnancy [21,22]. However, another study found the prevalence of anaemia in the third trimester to be 23.9%, which is still high [8]. We found the prevalence of anaemia to be 11.7% at 32–35 GWs and only 7.0% at ≥36 GWs, which might be related to the duration of the treatment for anaemia ranging from 24–27 GWs in our study. According to Chinese recommendations, all pregnant women should receive anaemia screening at the first antenatal visiting and again at 8–12 week intervals [15]. In our study, the prevalence of anaemia at 11–13 GWs, which is when most pregnant women attend the first antenatal visit, was only 3.4%. Meanwhile, we found the highest incidence of anaemia at 24–27 weeks, indicating that screening should be conducted earlier to detect the disease in a timely fashion.

Our results indicate that a history of anaemia, anaemia during a previous pregnancy, and multiple deliveries increased the risk of anaemia during pregnancy. These findings are consistent with those of other studies [9–11,23]. An Ethiopian longitudinal study showed that multiple pregnancies can reduce maternal Hb levels [24]. While the mechanism behind this was not elucidated, the results of a previous study suggest that multiparous women do not sufficiently recover from the burden of a prior pregnancy before having a child again [23]. Therefore, antenatal care visits and anaemia screening programmes should be more focused on and more frequent for multiparous women, especially those with a history of anaemia during a prior pregnancy or a history of anaemia in general. Even in pre-pregnancy stage, high risk women should get Hb and serum ferritin test to determine their iron status and ensure that prophylactic iron supplementation or similar treatment are delivered on time.

We also found a positive association of pre-pregnancy overweight and obesity with the incidence of maternal anaemia in the second or the third trimester. Research has reported that maternal obesity may negatively affect iron status during pregnancy through low-grade inflammation, a chronic state that may lead to anaemia [25,26]. However, other studies found no relationship between maternal BMI with iron deficiency and anaemia [27,28]. These divergences may be related to cross-study differences in participants and methods, but also to confounding factors such as access to medical care, food security status, or iron supplementation, *etc*. Here, we found the incidence of anaemia decreased in women with pre-pregnancy overweight and obesity. This may be explained by the women who are overweight or obese having higher iron stores or less plasma volume expansion. However, it could also be related to the fact that one-third of the subjects took iron supplementation prophylactically, or by the study hospitals being in urban areas, where the local population had the opportunity to maintain diverse or rich diets which included iron supplementation. Further research should explore the association between dietary intake during pregnancy and BMI.

### Strengths and limitations

To our knowledge, this is the first prospective multicentre study to investigate the incidence of anaemia in pregnant women at different pregnancy stages in China. However, our findings should be interpreted with some limitations in mind. First, we used an application through which pregnant women self-reported their data, and we excluded any women who did not return their data at all four check-ups. Second, we conducted our research in hospitals in more developed, urbanised Northern Chinese provinces, with the participants being highly educated and of relatively high socioeconomic status. This means that our results may not generalise to rural or lower socioeconomic populations in China, suggesting that they likely underestimate the true national prevalence. Third, due to concerns that the pregnant women could not accurately report their clinical data, we did not include questions supplemental iron and treatment information in the questionnaire. Fourth, since serum ferritin is not a routine test in study hospitals, we did not incorporate it in our analysis. Future research should collect data such as serum ferritin tests, inflammation markers, and supplemental iron and treatment information to better understand the prevalence and incidence of anaemia during pregnancy, and should also expand its scope to different economic areas and populations to ensure better generalisability.

## CONCLUSIONS

The overall prevalence and incidence of anaemia during pregnancy in our study were 23.2% and 20.5%, respectively, with both being the highest in the second trimester. Screening programmes in developed areas should attempt to capture women with a history of anaemia pre-pregnancy and a history of anaemia during prior pregnancy, and those have had multiple deliveries. Health education and counselling with respect to prenatal nutrition should be strengthened in general, both before and during pregnancy, especially for at-risk women. Further research should include serum ferritin to more precisely evaluate the evaluate the epidemiology of maternal anaemia, should extend to different economic areas and populations.

## Additional material


Online Supplementary Document
