# Supplementary material for: Incidence and factors on anaemia during pregnancy in China: a multicentre prospective cohort study
Source: J Glob Health. 2026 Feb 13;16:04058. doi: 10.7189/jogh.16.04058 (PMC12904026; doi:10.7189/jogh.16.04058)
Supplement: Online Supplementary Document [file jogh-16-04058-s001.pdf]

Supplement to: Zhang X, Wang X, Juan J, Gao D, Yang H, Zhang M, Chen X, Wang X, Ma Y, Teng Y, Zhang G, Wang Y, Meng H, Wang X, Yang Q, Xu L, Shan S. Incidence and factors on anaemia during pregnancy in China: a multicentre prospective cohort study. J Glob Health. 2026;16:04058.

STROBE Statement—checklist of items that should be included in reports of observational studies

	Item No.	Recommendation	Page No.	Relevant text from manuscript
**Title and abstract**	1	(*a*) Indicate the study's design with a commonly used term in the title or the abstract	1	Incidence and factors on anaemia during pregnancy in China: a multicentre prospective cohort study
		(*b*) Provide in the abstract an informative and balanced summary of what was done and what was found	2	Conclusions Both of prevalence and incidence of anaemia in pregnancy were higher in the second trimester. Targeted action is needed for women with a history of anaemia before or during a previous pregnancy and women who had multiple deliveries. Such efforts could include strengthened screening programs or better health education on prenatal nutrition during pregnancy and pre-pregnancy.
**Introduction**				
Background/rationale	2	Explain the scientific background and rationale for the investigation being reported	2-3	Therefore, to achieve the

				goal of lowering the prevalence of maternal anaemia below 10% by 2030 [12], China must implement screening programmes to identify high-risk women on time, before the occurrence of anaemia. However, most studies on maternal anaemia in the country were cross-sectional in design, with data on the incidence in each of the trimesters being especially lacking. Therefore, in this multicentre prospective cohort study, we sought to determine the incidence of anaemia during pregnancy at different gestational time points and the associated risk factors.
Objectives	3	State specific objectives, including any prespecified hypotheses	3	Therefore, in this multicentre prospective cohort study, we sought to determine the incidence of anaemia during pregnancy at different gestational time points and the associated risk factors.

Methods				
Study design	4	Present key elements of study design early in the paper	3	We conducted a prospective cohort study from October 2020 to June 2024 in 13 hospitals in seven provinces in North China: Beijing, Tianjin, Shannxi Province, Shanxi province, Shandong Province, Inner Mongolia, and Hebei Province
Setting	5	Describe the setting, locations, and relevant dates, including periods of recruitment, exposure, follow-up, and data collection	3-4	We conducted a prospective cohort study from October 2020 to June 2024 in 13 hospitals in seven provinces in North China: Beijing, Tianjin, Shannxi Province, Shanxi province, Shandong Province, Inner Mongolia, and Hebei Province.<br>**Participants**<br>We included pregnant women who had regular prenatal examinations and planned to be hospitalised for delivery, provided they were aged ≥18 years and consented to participant in the survey by signing an informed consent form. We excluded women with a pre-pregnancy history of

severe chronic diseases or pregnant women with severe mental illness.

Data collection process

We enrolled all eligible pregnant women in the first trimester at 11–13 weeks and followed them up three times in the second (24–27 gestational weeks (GWs)) and third (32–35 GWs) trimester, and once at ≥36 weeks. We collected their geographical location, history of anaemia, and Hb test results using the ‘91trial’ application, an electronic data collection platform we developed specifically for this project. In the first interview at 11–13 GWs, professional staff assisted the pregnant women download the ‘91trial’ application and share their data, as well as upload photographs of their laboratory test results, the data were including sociodemographic

characteristics included age, nationality, employment status, educational level and family income/month, the clinical characteristics included pre-pregnancy body mass index (BMI), gravidity, parity, history of anaemia and history of anaemia in previous pregnancies, other information included smoking before pregnancy, exposure to second-hand smoke before pregnancy , alcohol consumption before pregnancy, the frequency of coffee consumption, the frequency of tea consumption and use of supplemental iron . They then provided this information on their own at the three remaining time points, with reminders being sent to them to complete the survey. As all the study hospitals were tertiary hospitals with laboratory department overseen regularly in terms of quality control by

				the National centre for Clinical Laboratories, all laboratory tests were likewise conducted according to same standards and criteria.
Participants	6	(*a*) *Cohort study*—Give the eligibility criteria, and the sources and methods of selection of participants. Describe methods of follow-up<br>*Case-control study*—Give the eligibility criteria, and the sources and methods of case ascertainment and control selection. Give the rationale for the choice of cases and controls<br>*Cross-sectional study*—Give the eligibility criteria, and the sources and methods of selection of participants	3	**Participants**<br>We included pregnant women who had regular prenatal examinations and planned to be hospitalised for delivery, provided they were aged ≥18 years and consented to participant in the survey by signing an informed consent form. We excluded women with a pre-pregnancy history of severe chronic diseases or pregnant women with severe mental illness.<br>We enrolled all eligible pregnant women in the first trimester at 11–13 weeks and followed them up three times in the second (24–27 gestational weeks (GWs)) and third (32–35 GWs) trimester, and once at ≥36

weeks. We collected their geographical location, history of anaemia, and Hb test results using the '91trial' application, an electronic data collection platform we developed specifically for this project. In the first interview at 11–13 GWs, professional staff assisted the pregnant women download the '91trial' application and share their data, as well as upload photographs of their laboratory test results, the data were including sociodemographic characteristics included age, nationality, employment status, educational level and family income/month, the clinical characteristics included pre-pregnancy body mass index (BMI), gravidity, parity, history of anaemia and history of anaemia in previous pregnancies, other information included smoking before pregnancy, exposure to second-hand smoke before pregnancy , alcohol consumption before pregnancy, the frequency of coffee consumption, the

				frequency of tea consumption and use of supplemental iron . They then provided this information on their own at the three remaining time points, with reminders being sent to them to complete the survey. As all the study hospitals were tertiary hospitals with laboratory department overseen regularly in terms of quality control by the National centre for Clinical Laboratories, all laboratory tests were likewise conducted according to same standards and criteria.
		(*b*) *Cohort study*—For matched studies, give matching criteria and number of exposed and unexposed<br>*Case-control study*—For matched studies, give matching criteria and the number of controls per case	NA	
Variables	7	Clearly define all outcomes, exposures, predictors, potential confounders, and effect modifiers. Give diagnostic criteria, if applicable	4	We defined anaemia as a haemoglobin (Hb) values of <110 g/L and further categorised it as follows: mild anaemia (100–109 g/L), moderate anaemia (70–99 g/L) and severe anaemia(Hb <70 g/L) [14].<br>We calculated the overall incidence of anaemia:

numerator was the new cases of participants with anaemia at 24–27, 32–35 weeks, or ≥36 week,denominator was the number of women without anaemia at 11–13 weeks (Figure 1). Therefore, the total number of new cases of anaemia in the second or third trimester was 2176, and 10 607 pregnant women were not anaemic at 11–13 weeks; the overall incidence rate was 20.5% (n/N = 2176/10607).

Sociodemographic characteristics included age, nationality (Han and minority), employment status (‘yes’ or ‘no’), educational level (junior high school or below, senior high school, and university or above), and family income/month (RMB<3000, 3000–5000, 5000–10 000, and >10 000).

The clinical characteristics included pre-pregnancy body

				mass index (BMI), calculated as kg/m2 and categorised as underweight (<18.5), normal (18.5–23.9), overweight (24–27.9), obesity (≥28), according to the Chinese standard [15]; gravidity (0, 1 and ≥2); parity (0, 1 and ≥2); history of anaemia ('yes' or 'no'); and history of anaemia in previous pregnancies ('yes' or 'no').
Data sources/ measurement	8*	For each variable of interest, give sources of data and details of methods of assessment (measurement). Describe comparability of assessment methods if there is more than one group	*4-5*	We defined anaemia as a haemoglobin (Hb) values of <110 g/L and further categorised it as follows: mild anaemia (100–109 g/L), moderate anaemia (70–99 g/L) and severe anaemia(Hb <70 g/L) [14].<br><br>We calculated the overall incidence of anaemia: numerator was the new cases of participants with anaemia at 24–27, 32–35 weeks, or ≥36 week,denominator was the number of women without

anaemia at 11–13 weeks (Figure 1). Therefore, the total number of new cases of anaemia in the second or third trimester was 2176, and 10 607 pregnant women were not anaemic at 11–13 weeks; the overall incidence rate was 20.5% (n/N = 2176/10607).

Sociodemographic characteristics included age, nationality (Han and minority), employment status (‘yes’ or ‘no’), educational level (junior high school or below, senior high school, and university or above), and family income/month (RMB<3000, 3000–5000, 5000–10 000, and >10 000).

The clinical characteristics included pre-pregnancy body mass index (BMI), calculated as kg/m2 and categorised as underweight (<18.5), normal (18.5–23.9), overweight (24–27.9), obesity (≥28), according

				to the Chinese standard [15]; gravidity (0, 1 and ≥2); parity (0, 1 and ≥2); history of anaemia (‘yes’ or ‘no’); and history of anaemia in previous pregnancies (‘yes’ or ‘no’).<br>Other information included smoking before pregnancy (‘yes’ or ‘no’), exposure to second-hand smoke before pregnancy (‘yes’ or ‘no’), alcohol consumption before pregnancy (‘yes’ or ‘no’), the frequency of coffee consumption (‘everyday’, ‘1–2 days/week’, or ‘never’), the frequency of tea consumption (‘every day’, ‘1–2 days/week’, or ‘never’), and use of supplemental iron (‘yes’ or ‘no’).
Bias	9	Describe any efforts to address potential sources of bias	5	We also used multivariate logistic regression models for analysing association between incidence of anemia during pregnancy and variables, either unadjusted or adjusted for

				variables that were statistically significant in the univariate logistic regression models (age, education, BMI before pregnancy, history of anaemia, history of anaemia in previous pregnancies, history of gestation, and parity), and presented their outputs as odds ratios (ORs) and 95% confidence intervals (CIs)
Study size	10	Explain how the study size was arrived at	3	We included pregnant women who had regular prenatal examinations and planned to be hospitalised for delivery, provided they were aged ≥18 years and consented to participant in the survey by signing an informed consent form. We excluded women with a pre-pregnancy history of severe chronic diseases or pregnant women with severe mental illness.<br>Based on the relative research result, the prevalence of maternal anaemia in Chinese hospitals was 19.8% [13],

	α = 0.05, using PASS software version 15 (NCSS, LLC., USA) to calculate power ≥90%.

Continued on next page

Quantitative variables	11	Explain how quantitative variables were handled in the analyses. If applicable, describe which groupings were chosen and why	5	We presented categorical variables as numbers and percentages. Using Kolmogorov-Smirnova test combined with Q-Q plot to check for normality of continuous variables, if variables were normal distribution, then presented as means and standard deviations (SDs) . We performed multiple imputation for maternal age and all the statistics analysis using SPSS, version 26 (IBM, Armonk, New York, USA).
Statistical methods	12	(*a*) Describe all statistical methods, including those used to control for confounding		We constructed univariate logistic regression models to assess the associations between the incidence of anaemia during the second or third trimester and anaemia. We also used multivariate logistic regression models for analysing association between incidence of anemia during pregnancy and variables, either unadjusted or adjusted for variables that were statistically significant in the univariate logistic regression

			models (age, education, BMI before pregnancy, history of anaemia, history of anaemia in previous pregnancies, history of gestation, and parity), and presented their outputs as odds ratios (ORs) and 95% confidence intervals (CIs). We performed multiple imputation for maternal age and all the statistics analysis using SPSS, version 26 (IBM, Armonk, New York, USA).<br>All P-values are two-sided, with values <0.05 indicating statistical significance.
	(*b*) Describe any methods used to examine subgroups and interactions	NA	
	(*c*) Explain how missing data were addressed		We performed multiple imputation for maternal age and all the statistics analysis using SPSS, version 26 (IBM, Armonk, New York, USA).
	(*d*) *Cohort study*—If applicable, explain how loss to follow-up was addressed<br>*Case-control study*—If applicable, explain how matching of cases and controls was addressed<br>*Cross-sectional study*—If applicable, describe analytical methods taking account of sampling strategy	5	In total, 18 416 pregnant women attended the first interview at 13 hospitals, with 10 895 uploading all four Hb tests at 11 hospitals in five provinces: Beijing, Tianjin, Shanxi Province, Shandong Province, and Inner Mongolia.Figure1
	(*e*) Describe any sensitivity analyses	NA	
**Results**			

Participants	13*	(a) Report numbers of individuals at each stage of study—eg numbers potentially eligible, examined for eligibility, confirmed eligible, included in the study, completing follow-up, and analysed	5	In total, 18 416 pregnant women attended the first interview at 13 hospitals, with 10 895 uploading all four Hb tests at 11 hospitals in five provinces: Beijing, Tianjin, Shanxi Province, Shandong Province, and Inner Mongolia.Figure1
		(b) Give reasons for non-participation at each stage	5	In total, 18 416 pregnant women attended the first interview at 13 hospitals, with 10 895 uploading all four Hb tests at 11 hospitals in five provinces: Beijing, Tianjin, Shanxi Province, Shandong Province, and Inner Mongolia. See Figure1
		(c) Consider use of a flow diagram	13	Figure 1
Descriptive data	14*	(a) Give characteristics of study participants (eg demographic, clinical, social) and information on exposures and potential confounders	6-7	We found the prevalence of prevalence of anaemia during pregnancy in our sample to be 3.4% at 11–13 GWs, 15.2% at 24–27 GWs, 11.7% at 32–35 GWS, and 7.0% at ≥36 GWs. The overall prevalence of anaemia during pregnancy was 23.2%; its overall incidence in the second or third trimester was 20.5%, and was highest at 24–27 GWs (13.8%). Parity ≥2 times, a history of pre-pregnancy anaemia, and a history of anaemia in previous pregnancies

				were influencing factors for anaemia in the second or third trimester, while overweight and obesity before pregnancy also had positive association with of anaemia.
		(b) Indicate number of participants with missing data for each variable of interest	NA	
		(c) *Cohort study*—Summarise follow-up time (eg, average and total amount)	3	We conducted a prospective cohort study from October 2020 to June 2024 in 13 hospitals in seven provinces in North China: Beijing, Tianjin, Shannxi Province, Shanxi province, Shandong Province, Inner Mongolia, and Hebei Province.
Outcome data	15*	*Cohort study*—Report numbers of outcome events or summary measures over time	*6*	Prevalence of anaemia according to the GW of pregnancy<br>The mean Hb values were 127.35 g/L (SD = 10.34) (62 g/L–167 g/L) at 11–13 GWs, 118.02 g/L (SD = 8.86) (76 g/L–165 g/L) at 24–27 GWs, 119.87 g/L (SD = 9.26) (68 g/L–160 g/L) at 32–35 GWs, and 123.20 g/L (SD = 9.94) (69 g/L–167 g/L) at ≥36 GWs (Figure 2). In total, 378

pregnant women had anaemia at the first antenatal care interview, amounting to a prevalence of 3.4% (95% CI = 3.1–3.8%). The prevalence was the highest at 24–27 GWs (15.2%, 95% CI = 14.5–15.9%), followed by 32–35 GWs (11.7%; 95% CI = 11.1–12.3%) and ≥36 GWs (7.0%; 95% CI = 6.5–7.5%), respectively (Figure 2). The overall prevalence of anaemia during pregnancy was 23.2% (95% CI = 22.5–24.0%), and most anaemia cases were mild (Table 2).

Incidence of anaemia according to the GW

In the first antenatal interview at 11–13 GWs, 10 607 pregnant women were not anaemia. The incidence at 24–27 GWs (n/N = 1459/10 607), 32–35 GWs (n/N = 529/9148), and beyond 36 GWs (n/N = 188/8619) was 13.8% (95% CI = 13.1–14.4%), 5.8% (95% CI = 5.3–6.3%), and 2.2% (95% CI = 1.9–2.5%), respectively. The incidence in the second or third

				trimester (n/N = 2176/10 607) was 20.5% (95% CI = 19.8–21.3%) (Figure 1, Figure 2).
		Case-control study—Report numbers in each exposure category, or summary measures of exposure		
		Cross-sectional study—Report numbers of outcome events or summary measures		
Main results	16	(*a*) Give unadjusted estimates and, if applicable, confounder-adjusted estimates and their precision (eg, 95% confidence interval). Make clear which confounders were adjusted for and why they were included	15	Table 3
		(*b*) Report category boundaries when continuous variables were categorized		
		(*c*) If relevant, consider translating estimates of relative risk into absolute risk for a meaningful time period	NA	

Continued on next page

Other analyses	17	Report other analyses done—eg analyses of subgroups and interactions, and sensitivity analyses	NA	
**Discussion**				
Key results	18	Summarise key results with reference to study objectives	6-9	We found the prevalence of prevalence of anaemia during pregnancy in our sample to be 3.4% at 11–13 GWs, 15.2% at 24–27 GWs, 11.7% at 32–35 GWS, and 7.0% at ≥36 GWs. The overall prevalence of anaemia during pregnancy was 23.2%; its overall incidence in the second or third trimester was 20.5%, and was highest at 24–27 GWs (13.8%). Parity ≥2 times, a history of pre-pregnancy anaemia, and a history of anaemia in previous pregnancies were influencing factors for anaemia in the second or third trimester, while overweight and obesity before pregnancy also had positive association with of anaemia.
Limitations	19	Discuss limitations of the study, taking into account sources of potential bias or imprecision. Discuss both direction and magnitude of any potential bias	9	First, we used an application through which pregnant women self-reported their data, so

excluding pregnant women missed part of information besides HB results. Second, we conducted our research in hospitals in more developed, urbanised Northern Chinese provinces, with the participants being highly educated and of relatively high socioeconomic status. This means that our results may not generalise to rural or lower socioeconomic populations in China, suggesting that they likely underestimate the true national prevalence. Third, due to concerns with respect to the subjectivity among the pregnant women during the collection of information, inquiries regarding supplemental iron and treatment information were not included in the questionnaire. Fourth, since serum ferritin is not a routine test in study hospitals, we did not incorporate it in our analysis. Future research should collect data such as serum ferritin tests, inflammation markers, and supplemental iron and

				treatment information to better understand the prevalence and incidence of anaemia during pregnancy, and should also expand its scope to different economic areas and populations to ensure better generalisability.
Interpretation	20	Give a cautious overall interpretation of results considering objectives, limitations, multiplicity of analyses, results from similar studies, and other relevant evidence	9	The overall prevalence and incidence of anaemia during pregnancy in our study were 23.2% and 20.5%, respectively, with both being the highest in the second trimester. Screening programmes in developed areas should attempt to capture women with a history of anaemia pre-pregnancy and anemia history during previoud pregnancy, and those have had multiple deliveries. Health education and counselling with respect to prenatal nutrition should be strengthened in general, both before and during pregnancy, especially for at-risk women. Further research should include serum ferritin to more precisely evaluate the evaluate the epidemiology of maternal anaemia, should extend to different economic areas and populations.

Generalisability	21	Discuss the generalisability (external validity) of the study results	9	Second, we conducted our research in hospitals in more developed, urbanised Northern Chinese provinces, with the participants being highly educated and of relatively high socioeconomic status. This means that our results may not generalise to rural or lower socioeconomic populations in China, suggesting that they likely underestimate the true national prevalence
**Other information**				
Funding	22	Give the source of funding and the role of the funders for the present study and, if applicable, for the original study on which the present article is based	13	This work was supported by grants from the Chinese Preventive Medicine Association

*Give information separately for cases and controls in case-control studies and, if applicable, for exposed and unexposed groups in cohort and cross-sectional studies.

Note: An Explanation and Elaboration article discusses each checklist item and gives methodological background and published examples of transparent reporting. The STROBE checklist is best used in conjunction with this article (freely available on the Web sites of PLoS Medicine at http://www.plosmedicine.org/, Annals of Internal Medicine at http://www.annals.org/, and Epidemiology at http://www.epidem.com/). Information on the STROBE Initiative is available at www.strobe-statement.org.
